# Optimization and Characterization of P(EDOT-*co*-Th)-Incorporated Poly(acrylamide)/Poly(vinyl alcohol) Conductive Hydrogels

**DOI:** 10.3390/mi17050603

**Published:** 2026-05-14

**Authors:** Kai-Wei Huang, Chun Hao Wang, Chien-Yin Lin, Rajan Deepan Chakravarthy, Hsin-Yu Liu, Yu-Hsu Chen, Mei-Yu Yeh, Hsin-Chieh Lin

**Affiliations:** 1 Department of Chemistry, Chung Yuan Christian University, Taoyuan 320314, Taiwan; 2 Neurosurgical Department, Taoyuan General Hospital, Ministry of Health and Welfare, Taoyuan 330215, Taiwan; 3 Department of Materials Science and Engineering, National Yang Ming Chiao Tung University, Hsinchu 300093, Taiwan; deepannycu@gmail.com; 4 Department of Orthopedic Surgery, Taoyuan General Hospital, Ministry of Health and Welfare, Taoyuan 330215, Taiwan; magister.yuhsu@gmail.com; 5 Center for Intelligent Drug Systems and Smart Bio-Devices (IDS^2^B), National Yang Ming Chiao Tung University, Hsinchu 30068, Taiwan

**Keywords:** conductive hydrogels, functionalized poly(vinyl alcohol), conductive polymers, adhesive hydrogels

## Abstract

Conductive hydrogels are functional materials that combine soft, highly hydrated properties with electrical signal transmission capabilities. Their conductivity arises from ionic or electronic pathways, and the key design challenge is achieving good conductivity and long-term stability without compromising mechanical performance and biocompatibility. Among various conductive components, conductive polymers have attracted considerable attention due to their tunable mechanical properties, high electrical conductivity, good biocompatibility, and facile synthesis routes. In this study, a series of conductive hydrogels were rationally designed and fabricated by copolymerizing acrylamide and *N*,*N*′-methylenebisacrylamide with functionalized poly(vinyl alcohol) (PVA) and poly(3,4-ethylenedioxythiophene-*co*-thiophene) [P(EDOT-*co*-Th)]. The functionalized PVA provided multiple dynamic hydrogen-bonding sites, significantly enhancing the toughness of the hydrogel and its adhesion to various substrates, while the P(EDOT-*co*-Th) copolymer imparted good and stable electrical conductivity. By systematically adjusting the amount of functionalized PVA, the mechanical strength, adhesiveness, and durability of the conductive hydrogels were effectively optimized. The optimized hydrogel exhibited robust adhesion to a wide range of surfaces, excellent fatigue resistance, and long-term stability under repeated mechanical deformation. Moreover, the combination of mechanical resilience and good conductivity enabled precise and reliable signal transduction, highlighting its strong potential as a next-generation material for wearable strain and pressure sensors.

## 1. Introduction

Conductive hydrogels are a class of functional materials that combine softness, high water content, and electrical signal transmission capability [1,2,3,4,5,6]. Owing to their three-dimensional polymer network structures containing large amounts of water, these materials exhibit tissue-like softness and excellent deformability. Therefore, they have attracted significant attention in fields such as flexible electronics, wearable sensors, biomedical monitoring, and human motion detection [7,8,9,10]. Compared with traditional rigid electronic materials, conductive hydrogels can maintain good electrical conductivity while undergoing large deformations such as bending, stretching, and compression [11,12]. This property makes them particularly suitable for sensing devices that need to conform to the human body surface, such as those used for monitoring joint movement, finger bending, or muscle activity. However, several challenges still exist in the design of conductive hydrogels. One of the most critical issues is how to maintain high conductivity and long-term stability without compromising the mechanical properties, stretchability, and biocompatibility of the material. Therefore, developing conductive hydrogels that simultaneously possess high stretchability, stable conductivity, and strong adhesion has become an important research direction in materials science and bioelectronics in recent years.

The conductive mechanisms of hydrogels can generally be classified into ionic conduction and electronic conduction [13,14,15,16,17]. Ionic conductive hydrogels rely on ions dissolved in the aqueous phase to transport electrical charges. However, such materials may suffer from performance degradation during long-term operation due to water evaporation or ion leakage, which reduces their conductivity. In contrast, electronic conductive networks constructed by conductive polymers exhibit better stability and higher conductivity, making them an increasingly important strategy for developing conductive hydrogels. Among the various conductive components, conductive polymers have attracted extensive attention because of their tunable mechanical properties, high electrical conductivity, good biocompatibility, and relatively simple synthesis methods [18,19,20,21,22,23]. Common conductive polymers such as polypyrrole (PPy), polyaniline (PANI), and poly(3,4-ethylenedioxythiophene) (PEDOT) possess conjugated electronic structures that can form stable electron-transport pathways within materials, enabling hydrogels to retain electrical conductivity while maintaining their soft characteristics. Furthermore, incorporating conductive polymers into polymer matrices can not only enhance electrical performance but also improve mechanical strength and stability, making them more suitable for applications in flexible electronics and wearable sensing systems [24,25].

On the other hand, to further enhance the mechanical performance and structural stability of hydrogels, researchers often introduce crosslinked structures and multiple intermolecular interactions into the polymer network. For example, acrylamide (AAM) is widely used to prepare polyacrylamide hydrogels because of its good water solubility and polymerization ability [26,27]. When combined with *N*,*N*′-methylenebisacrylamide (MBAA) as a crosslinking agent, a stable three-dimensional polymer network can be formed [28]. This crosslinked structure provides the fundamental mechanical support for hydrogels, enabling them to maintain structural integrity during stretching. Nevertheless, single-polyacrylamide hydrogels may still exhibit insufficient toughness under high-strain conditions, prompting researchers to introduce additional polymer components to improve their mechanical properties.

Among these materials, poly(vinyl alcohol) (PVA) is a polymer with excellent biocompatibility and mechanical performance [29,30,31]. The PVA molecular chain contains abundant hydroxyl (–OH) functional groups that can form extensive hydrogen bonding interactions with other polymer chains. These interactions help enhance the toughness and mechanical stability of hydrogels. Moreover, the hydroxyl groups in PVA can also interact with the surfaces of different materials through hydrogen bonding or other intermolecular forces, providing hydrogels with good adhesive properties. By integrating PVA with conductive polymers within the hydrogel network, it becomes possible to fabricate multifunctional conductive hydrogels that exhibit high stretchability, stable conductivity, and strong adhesion.

Based on the above research background, in this study a series of conductive hydrogels were successfully prepared by constructing a crosslinked polymer network using AAM and MBAA, combined with functionalized PVA and a conductive copolymer of poly(3,4-ethylenedioxythiophene-*co*-thiophene) [P(EDOT-*co*-Th)]. Through the regulation of material composition and network structure, the resulting hydrogels exhibited both excellent mechanical properties and stable electrical conductivity. Their chemical structures were analyzed using Fourier-transform infrared spectroscopy (FTIR), while their mechanical properties, electrochemical impedance characteristics, and strain-sensing performance were systematically investigated. The potential application of the prepared hydrogels in flexible strain sensors was also explored. The experimental results demonstrate that the developed hydrogels possess outstanding stretchability, electrical conductivity, and adhesion, indicating their promising potential for applications in wearable electronics and human motion monitoring systems.

## 2. Materials and Methods

### 2.1. Materials and Method

Acrylamide (AAM) was supplied by Acros Organics. *N*,*N*′-Methylene-bisacrylamide (MBAA) and poly(vinyl alcohol) (PVA, average molecular weight 10,000–26,000) were obtained from Alfa Aesar (Ward Hill, MA, USA). 4-Formylbenzoic acid, 4-(dimethylamino)pyridine (DMAP), *N*,*N*′-dicyclohexylcarbodiimide (DCC), and potassium persulfate were purchased from Sigma-Aldrich (St. Louis, MO, USA). 3,4-Ethylenedioxythiophene (EDOT) was acquired from Tokyo Chemical Industry (Tokyo, Japan), while thiophene (Th) was obtained from Alfa Aesar. All reagents were used as received without further purification.

The synthesis of functionalized PVA was performed according to a reported method [32]. Briefly, PVA (5.000 g, 0.048 mmol) was vacuum-dried and dissolved in DMSO at 90 °C under a nitrogen atmosphere with continuous stirring until a clear solution was obtained. 4-Formylbenzoic acid (11.260 g, 0.075 mol) was then added, and the reaction temperature was reduced to 50 °C. Subsequently, DCC (1.000 g, 0.0048 mol) and DMAP (0.100 g, 0.001 mol) were added under nitrogen, and the reaction was allowed to proceed for 18 h. The resulting precipitate was washed with cold acetone and further purified by dialysis at 50 °C for 72 h. Finally, the product was freeze-dried for 72 h to obtain the solid product (APVA). P(EDOT-*co*-Th) was synthesized following our previously reported method, with an EDOT: Th molar ratio of 9:1 [33].

### 2.2. Preparation of Hydrogels

A series of hydrogels (CH-1 to CH-4) were prepared *via* free-radical polymerization. For CH-1, AAM (1.13 g) was dissolved in 2.5 mL of deionized water, followed by the sequential addition of MBAA (2.50 mg), P(EDOT-*co*-Th) copolymer (5.00 mg), and APVA (30.00 mg). The mixture was stirred at room temperature for 5 min, after which potassium persulfate (3.75 mg) was added as the initiator. The solution was then heated to 70 °C and allowed to react for 2 h to obtain CH-1 hydrogel. CH-2, CH-3, and CH-4 hydrogels were prepared following the same procedure, with the APVA content adjusted to 40.00 mg, 50.00 mg, and 60.00 mg, respectively, while all other reaction conditions were maintained.

### 2.3. Characterizations

Functional group analysis of the hydrogel samples was performed using Fourier-transform infrared (FTIR) spectroscopy on a Nicolet iS5 spectrometer (Thermo Fisher Scientific, Waltham, MA, USA). The spectra were recorded over the wavenumber range of 4000–400 cm^−1^ at room temperature to identify the characteristic functional groups and confirm the formation of the polymer network.

Electrochemical measurements were conducted using a CHI627E electrochemical workstation (CH Instruments, Austin, TX, USA). The hydrogel samples used for strain sensing were prepared in a circular shape with a diameter of 23 mm and a thickness of 0.5 mm. Electrochemical impedance spectroscopy (EIS) was carried out to evaluate the electrical conductivity of the hydrogels over an appropriate frequency range under ambient conditions. A sandwich-structured electrochemical cell was constructed by placing the conductive hydrogel sheet between two indium tin oxide (ITO) glass electrodes. The hydrogel sample had dimensions of 2.5 mm × 2.5 mm × 0.5 mm (length × width × thickness). The electrical conductivity (σ, S/m) was calculated using the following equation:

$$\sigma =\;\frac{L}{R\;\times \;A}$$

where *L* is the distance between the two ITO electrodes, *R* is the measured resistance, and *A* is the cross-sectional area of the hydrogel sample.

Mechanical properties of the hydrogels were evaluated using a Gotech AI-3000-U (Taichung, Taiwan) universal tensile testing machine. The hydrogel samples were cut into rectangular specimens with uniform dimensions (length: 20 mm, width: 10 mm, thickness: 2 mm), and tensile tests were performed at a constant stretching rate (100 mm/min). The stress–strain curves were recorded to determine tensile strength, elongation at break, and toughness of the hydrogels. The experiments were conducted in triplicate.

## 3. Results and Discussion

### 3.1. Hydrogel Preparation and Mechanical Characterization

**Figure 1:**
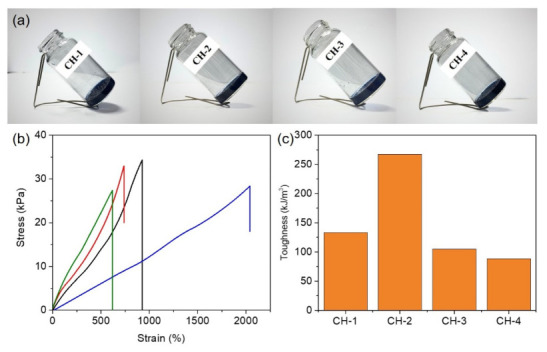
(**a**) Optical images of CH-1, CH-2, CH-3, and CH-4 hydrogels. (**b**) Tensile stress–strain curves of CH-1 to CH-4 hydrogels (CH-1, black; CH-2, blue; CH-3, red; CH-4, green). (**c**) Toughness of CH-1 to CH-4 hydrogels.

In this work, conductive hydrogels were synthesized *via* copolymerization of AAM and MBAA in the presence of functionalized PVA (APVA) and P(EDOT-*co*-Th). By tuning the APVA content, a series of hydrogels labeled CH-1, CH-2, CH-3, and CH-4 were obtained (Figure 1a). This material design strategy integrates the regulation of polymer network structures with the incorporation of conductive polymers, enabling the resulting hydrogels to simultaneously exhibit excellent mechanical properties and stable electrical conductivity, thereby providing a promising material platform for applications in flexible electronics and smart sensing devices. To evaluate the mechanical properties of the prepared conductive hydrogels, tensile stress–strain tests were performed on the four samples, CH-1, CH-2, CH-3, and CH-4, as shown in Figure 1b. The results indicate that hydrogels with different compositions exhibit distinct mechanical strength and stretchability. The maximum tensile stresses of CH-1, CH-2, CH-3, and CH-4 were 34.4 ± 1.1 kPa, 28.4 ± 0.4 kPa, 33.1 ± 0.6 kPa, and 27.1 ± 1.3 kPa, respectively. In terms of maximum strain, the samples demonstrated varying degrees of stretchability. The maximum strains of CH-1, CH-2, CH-3, and CH-4 were 926 ± 35%, 2037 ± 61%, 736 ± 57%, and 614 ± 83%, respectively. Among them, CH-2 exhibited the most outstanding stretchability, with a strain reaching 2037%, indicating that the material can undergo deformation exceeding 20 times its original length without fracture. Such high stretchability is particularly important for flexible materials because wearable electronic devices and human motion sensors often experience repeated bending, stretching, and twisting during operation. Therefore, high-strain tolerance can significantly enhance the stability and durability of materials in dynamic environments. In addition to strength and stretchability, the toughness of the material is another critical parameter for evaluating the mechanical performance of hydrogels. Toughness is typically represented by the area under the stress–strain curve, which reflects the amount of energy that a material can absorb before fracture. The calculated toughness values for CH-1, CH-2, CH-3, and CH-4 were 133.28, 267.39, 105.00, and 88.60 kJ/m^3^, respectively (Figure 1c). From these results, CH-2 exhibited significantly higher toughness than the other samples, indicating that it can absorb more mechanical energy during stretching without failure. This superior toughness mainly arises from its high stretchability, which allows the material to dissipate mechanical energy through large deformation under external stress. Additionally, to systematically evaluate the contributions of the conductive component and polymer modification to the overall performance of the hydrogels, CH-0, Control-1, and Control-2 were prepared (detailed procedures are provided in the Supplementary Materials). As shown in Table S1 and Figure S1, in the absence of APVA, the tensile strength and strain were 18.9 ± 0.3 kPa and 146 ± 19%, respectively, indicating that the incorporation of APVA significantly enhances both strength and stretchability (CH-0). In contrast, the absence of P(EDOT-co-Th) or the use of unmodified PVA led to a marked reduction in the stretchability of the hydrogels (Control-1 and Control-2).

### 3.2. Spectroscopic and Electrochemical Analysis of Hydrogels

**Figure 2:**
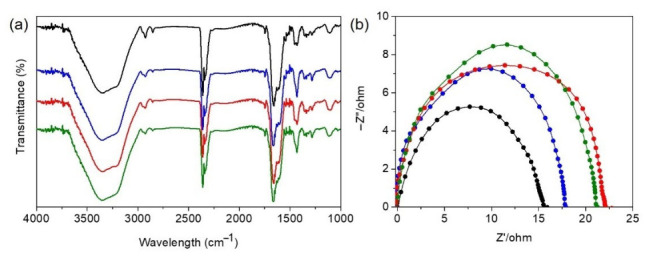
(**a**) FTIR spectra; (**b**) EIS spectra of hydrogels CH-1–CH-4 (CH-1, black; CH-2, blue; CH-3, red; CH-4, green).

To analyze the chemical structure and functional group composition of the prepared hydrogel materials, Fourier-Transform Infrared Spectroscopy (FTIR) was employed for characterization. As shown in the FTIR spectra in Figure 2a, CH-1, CH-2, CH-3, and CH-4 exhibit several representative absorption peaks across different wavenumber regions, indicating the presence of characteristic functional groups in the materials. First, a broad and prominent absorption band can be observed in the 3200–3500 cm^−1^ region, which corresponds to the stretching vibrations of –OH and –NH_2_ groups [34,35,36,37]. The broad nature of this peak indicates the presence of strong hydrogen bonding interactions within the material, such as hydrogen bonding between the hydroxyl groups of PVA and the polyacrylamide chains [38]. These intermolecular interactions contribute to improving the mechanical stability and toughness of the hydrogel. A distinct absorption peak is observed around 1650 cm^−1^, which corresponds to the C=O stretching vibration. This peak is a characteristic signal of the polyacrylamide structure formed by AAM and MBAA [39,40]. In the 1500–1600 cm^−1^ region, absorption signals associated with C=C conjugated structure vibrations can be observed. These signals are mainly attributed to the conjugated double bonds in the thiophene rings of the conductive polymer P(EDOT-*co*-Th) [33,41]. Furthermore, absorption peaks corresponding to C–S bond vibrations can be observed in the 1200–1400 cm^−1^ region [33,41]. This signal is an important characteristic of the thiophene ring structure and further confirms the presence of P(EDOT-*co*-Th) within the hydrogel matrix. Meanwhile, a C–O stretching vibration peak can be observed around 1000–1150 cm^−1^, which mainly originates from the alcohol C–O bonds in the PVA molecular chains, and may also partially arise from the C–O–C structure in the EDOT unit [33,34,40]. The FTIR spectra reveal the coexistence of several characteristic absorption peaks, including –OH/–NH_2_, C=O, C=C, C–S, and C–O, which correspond to the structural signals of PVA, AAM/MBAA, and P(EDOT-*co*-Th), respectively. The simultaneous presence of these characteristic peaks demonstrates that all components have been successfully integrated into the same polymer network, confirming the successful formation of a conductive composite hydrogel composed of AAM, MBAA, PVA, and P(EDOT-*co*-Th). Moreover, scanning electron microscopy (SEM) was performed on CH-1 to CH-4 hydrogels (Figure S2). The SEM images show that CH-1 exhibits a dense and compact layered structure, indicating a tightly packed polymer network. With increasing APVA content (CH-2 to CH-4), the microstructure becomes more heterogeneous and interconnected, forming a porous and hierarchical network. Notably, CH-2 displays a well-balanced and uniformly interconnected structure, which is advantageous for maintaining mechanical integrity. This structural evolution supports the formation of a composite polymer network. The optimized network in CH-2 correlates with its superior mechanical properties, including high stretchability and toughness. In contrast, excessive or insufficient network density may lead to reduced mechanical performance due to structural inhomogeneity or weakened connectivity. This structural confirmation provides an important foundation for the mechanical performance of the material.

Figure 2b presents the Electrochemical Impedance Spectroscopy (EIS) results of the hydrogel samples [42,43]. From the figure, the impedance values of CH-1, CH-2, CH-3, and CH-4 are 15 ± 1.3, 18 ± 2.6, 22 ± 1.7, and 21 ± 3.1 Ω, respectively. The electrical properties of the hydrogels were evaluated by calculating both the resistivity and conductivity based on the measured resistance values and sample dimensions (Table S2). The resistivity values of CH-1, CH-2, CH-3, and CH-4 were determined to be 0.1875, 0.225, 0.275, and 0.2625 Ω·m, respectively. Correspondingly, the calculated conductivities were 5.33, 4.44, 3.64, and 3.81 S/m. An overall trend can be observed in which the impedance of the hydrogel gradually increases with increasing PVA content in the formulation. Consistently, it can be found that the electrical conductivity decreases as the PVA content increases. This phenomenon may be attributed to the fact that PVA is an electrically insulating polymer, and an increase in its content may partially interfere with the conductive pathways formed by the conductive polymer network. As a result, the electron or ion transport pathways become partially hindered, leading to an increase in the overall electrical impedance of the material. In addition, the hydrogen bonding interactions formed between PVA molecular chains may make the polymer network structure more compact, which can further reduce the continuity of the conductive network. However, electrical conductivity is not the only factor used to evaluate the application potential of hydrogels; mechanical performance is equally important. By comprehensively comparing the stress, strain, toughness, and conductivity of the different samples, it can be observed that a certain degree of performance trade-off exists among the various formulations. After overall evaluation, CH-2 exhibits the most outstanding stretchability and toughness while still maintaining good electrical conductivity, allowing it to achieve an optimal balance between mechanical and electrical properties. Therefore, CH-2 was ultimately selected as the optimal candidate for subsequent hydrogel strain sensor application tests and performance evaluation in this study. Moreover, compared with previously reported systems, CH-2 exhibits superior stretchability and competitive electrical conductivity, highlighting its advantageous balance between mechanical and electrical performance (Table S3) [44,45,46,47,48,49,50,51].

### 3.3. Electrical Conductivity and Damage Recovery Performance of CH-2 Hydrogel

**Figure 3:**
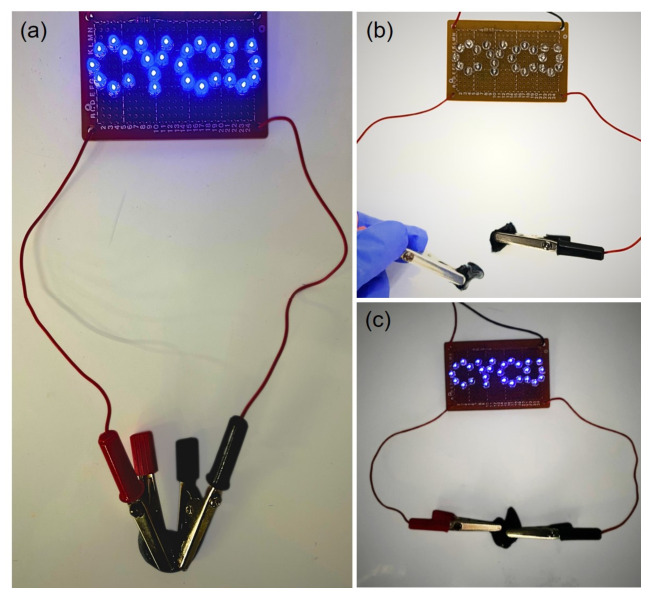
Demonstration of electrical conductivity and damage recovery of the CH-2 hydrogel. (**a**) LED lighting test using the CH-2 hydrogel as a conductive bridge to complete the circuit. (**b**) The LED turned off after the hydrogel was cut, indicating the conductive pathway was broken. (**c**) The LED was re-illuminated after the separated hydrogel pieces were reconnected, demonstrating the recovery of electrical conductivity.

To verify the electrical conductivity of the CH-2 hydrogel, a simple and intuitive LED lighting test was conducted [52]. LED demonstrations are commonly used as a preliminary method for evaluating conductive hydrogels or flexible conductive materials because the illumination of the LED and changes in its brightness can quickly indicate whether the material possesses stable current transmission capability. As shown in Figure 3a, multiple LEDs were arranged to form the letters “CYCU”, and the CH-2 hydrogel was used as a conductive bridge to connect the circuit. When the power supply was turned on, the LEDs were successfully illuminated and emitted bright light. This observation indicates that an effective conductive pathway had formed within the CH-2 hydrogel, allowing electric current to pass through the material smoothly and confirming its good electrical conductivity. As a control, Control-1 without P(EDOT-*co*-Th) was unable to light the LED (Figure S3). To further evaluate the conductive stability and recoverability of the material under mechanical damage, a mechanical destruction test was performed on the CH-2 hydrogel. As shown in Figure 3b, the hydrogel was first cut or physically damaged, interrupting the conductive pathway. At this stage, the LED immediately turned off, indicating that the circuit could no longer conduct electricity. Subsequently, the damaged pieces of the CH-2 hydrogel were reconnected, as illustrated in Figure 3c. After reconnection, the hydrogel was reintroduced into the circuit, and the LED was illuminated again, demonstrating that electrical current could once again pass through the hydrogel material. This result indicates that the CH-2 hydrogel retains a certain degree of recoverable conductivity even after mechanical damage. It is worth noting that although the LED was able to emit light after reconnection, its brightness was slightly lower compared with the original undamaged state. This phenomenon may be attributed to partial disruption of the internal conductive network during the cutting and reconnection processes, which slightly reduced the continuity of the conductive pathways. As a result, the overall electrical resistance increased and the efficiency of current transmission decreased. Nevertheless, the LED remained clearly illuminated, indicating that the hydrogel still maintained a relatively good level of electrical conductivity. These results demonstrate that the CH-2 hydrogel not only exhibits stable conductive performance but can also maintain a certain level of functionality after mechanical damage. To further investigate the recovery behavior of the CH-2 hydrogel, rheological recovery tests were conducted (Figure S4). The strain-dependent behavior revealed an intersection of the storage modulus (G′) and loss modulus (G″) at a strain of approximately 1878%, indicating a transition from elastic-dominated to viscous-dominated behavior. Under alternating step-strain conditions (10% and 2000%), cyclic measurements of G′ and G″ were performed. At a low strain of 10%, G′ remained higher than G″, demonstrating the predominance of elastic behavior and the integrity of the network structure. In contrast, at a high strain of 2000%, G″ exceeded G′, indicating disruption of the network and a transition to viscous behavior. Notably, upon returning to low strain, G′ rapidly recovered and again surpassed G″. This reversible transition was consistently observed over three consecutive cycles, with minimal loss in modulus, demonstrating excellent recovery efficiency. These results indicate that the internal network structure can be dynamically disrupted and reformed, which is attributed to the presence of dynamic hydrogen-bonding interactions within the hydrogel network. Such characteristics are particularly important for flexible electronic devices and strain sensors, because materials used in these applications often experience repeated stretching, bending, or localized damage during practical use. Therefore, materials with recoverable conductivity can effectively enhance the reliability, durability, and service life of flexible electronic devices.

### 3.4. Strain-Sensing Performance of the CH-2 Hydrogel

**Figure 4:**
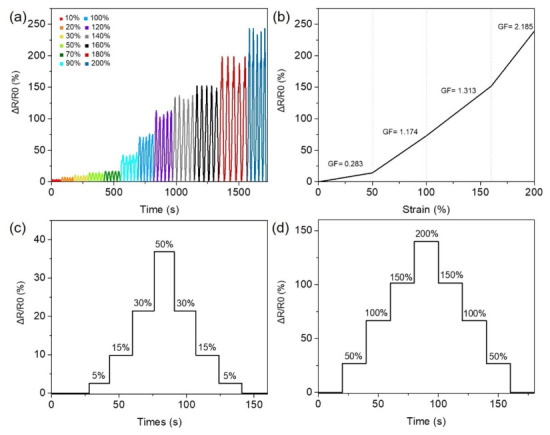
(**a**) Cyclic resistance response of the CH-2 strain sensor at different applied strains (10%, 20%, 30%, 50%, 70%, 90%, 100%, 120%, 140%, 160%, 180%, and 200%). (**b**) Gauge factor (GF) values of the CH-2 sensor. (**c**,**d**) Relative resistance change as a function of stretching and releasing.

In the performance evaluation of the strain sensor, the CH-2 hydrogel was used as the sensing material in this study, and its resistance variation behavior was systematically tested and analyzed to investigate its sensing capability and stability under different deformation conditions [53,54,55,56]. Since strain sensors in practical applications must be able to detect both small deformations and large-scale motions, multiple strain ranges were designed in the testing process to comprehensively evaluate the sensing performance of the CH-2 hydrogel sensor. The test results are shown in Figure 4a,b. Within the strain range of 10% to 200%, the CH-2 hydrogel strain sensor exhibited distinct and repeatable resistance response signals, demonstrating good strain responsiveness and a stable conductive structure. Even under relatively large stretching conditions, the sensing signal maintained a regular and stable variation trend, indicating that the conductive pathways within the material remained continuous during deformation. To further quantify the sensitivity of the sensor to strain, the Gauge Factor (GF) was calculated. The GF value is an important parameter used to evaluate the performance of strain sensors and is defined as the ratio between the relative resistance change and the applied strain [56]. The calculation results show that the maximum GF value of the CH-2 hydrogel sensor reached 2.185, indicating that the material can produce significant resistance changes during deformation and thus possesses good sensing sensitivity. Furthermore, to verify the stability and repeatability of the sensor under different strain conditions, a tensile testing machine was used to precisely set multiple cyclic strain conditions for evaluation (Figure 4c,d). First, a strain sequence of 5%, 15%, 30%, 50%, 30%, 15%, and 5% was applied to simulate a deformation process in which the strain gradually increases and then decreases. The results show that the resistance signal of the sensor changed correspondingly with the increase and decrease in strain, and the sensor maintained stable signal output during repeated cycles. Subsequently, larger strain conditions of 50%, 100%, 150%, 200%, 150%, 100%, and 50% were further applied to evaluate the sensor performance under significant stretching deformation. The results demonstrate that even under high-strain conditions, the CH-2 hydrogel maintained good sensing responsiveness and stable signal variation. These results indicate that the CH-2 hydrogel strain sensor exhibits stable, repeatable, and sensitive resistance response characteristics over a wide strain range. The combination of high stretchability and stable electrical conductivity provides this material with strong potential for applications in wearable electronics, human motion monitoring, and intelligent health sensing systems.

**Figure 5:**
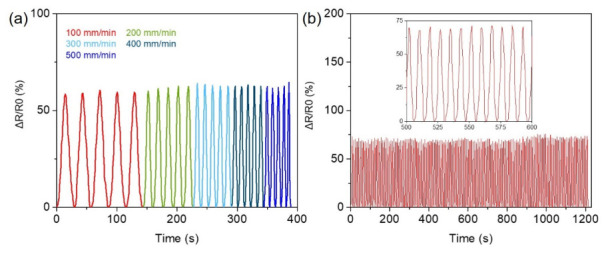
(**a**) Relative resistance response of the CH-2 hydrogel at different stretching speeds (100, 200, 300, 400, and 500 mm/min). (**b**) Relative resistance change in the CH-2 hydrogel during loading–unloading cycles over 1200 s.

In addition to evaluating the influence of different strain magnitudes on the resistance signals of the sensor, this study further investigated the sensing performance of the CH-2 hydrogel under different strain frequencies, as well as its responsiveness to external deformation. In practical applications, wearable strain sensors often encounter motions at different speeds, such as slow joint bending or rapid hand movements. Therefore, whether the sensing material can maintain stable signal output under different stretching rates is crucial for its practical applicability. As shown in Figure 5a, a tensile testing machine was used to apply different stretching rates. The stretching speeds were sequentially set from slow to fast at 100 mm/min, 200 mm/min, 300 mm/min, 400 mm/min, and 500 mm/min, in order to simulate deformation conditions caused by human motions at various speeds. The results demonstrate that under these different strain rates, the CH-2 hydrogel sensor produced clear and regular resistance response signals. Even at higher stretching speeds, the resistance signals maintained good repeatability and stability, without significant signal distortion or irregular fluctuations. These results indicate that the conductive network within the CH-2 hydrogel remains structurally intact during dynamic stretching, allowing the electron transport pathways to change predictably with the deformation of the material. Consequently, the CH-2 hydrogel exhibits reliable sensing stability and performance under different strain frequencies and deformation speeds, making it suitable for dynamic motion detection in wearable sensing applications. Furthermore, the long-term operational stability and durability of the CH-2 hydrogel sensor were also evaluated. As shown in Figure 5b, a continuous cyclic stretching test was conducted for 1200 s, with a fixed strain of 100% applied repeatedly to simulate the operating conditions that the sensor may experience during prolonged practical use. During the entire testing process, the sensor underwent repeated stretching and releasing cycles while its resistance signal was continuously monitored in real time. The results show that throughout the 1200-s continuous cyclic test, the resistance signal of the CH-2 hydrogel sensor remained stable and highly repeatable, and no significant signal attenuation or baseline drift was observed between cycles. This outcome demonstrates that the CH-2 hydrogel can maintain a stable conductive structure and sensing performance even under long-term repeated deformation conditions, indicating excellent mechanical durability and signal stability. These characteristics further highlight the strong potential of the CH-2 hydrogel for long-term wearable sensing applications and flexible electronic devices.

### 3.5. Adhesion Performance of the CH-2 Hydrogel

**Figure 6:**
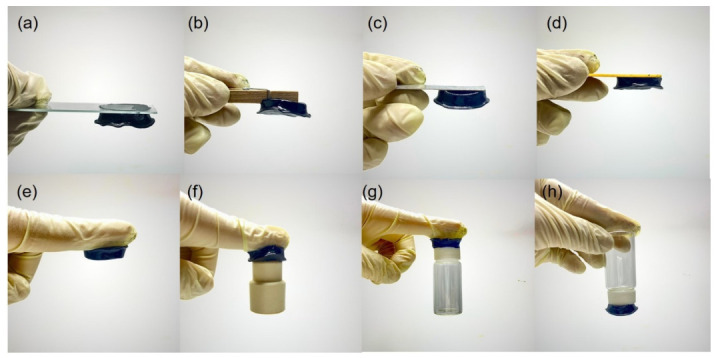
Adhesion images of the CH-2 hydrogel on different substrates: (**a**) glass, (**b**) wood, (**c**) aluminum sheets, (**d**) copper sheets, (**e**) gloves, (**f**) rubber, and (**g**,**h**) plastic.

Finally, the surface adhesion properties of the CH-2 hydrogel were evaluated to investigate its ability to adhere to different material surfaces. Good adhesion is particularly important for the practical application of hydrogel-based strain sensors [57,58,59]. As shown in Figure 6, several substrates with different surface properties were selected for testing, including glass, wood, aluminum sheets, copper sheets, gloves, rubber, and plastic, in order to comprehensively evaluate the adhesion performance of the CH-2 hydrogel. The experimental results demonstrate that the CH-2 hydrogel exhibits excellent adhesion on all of these material surfaces. After contacting the substrates, the hydrogel could firmly adhere to their surfaces without easily detaching, indicating its strong interfacial adhesion capability. This broad adhesion ability is mainly attributed to the multiple hydrogen-bonding interactions formed by PVA and polyacrylamide chains within the hydrogel network. The PVA molecular chains contain abundant hydroxyl groups (–OH), while the polyacrylamide structure contains amide groups (–CONH_2_). These functional groups can interact with different substrate surfaces through hydrogen bonding, van der Waals forces, or other intermolecular interactions, thereby enhancing the adhesion between the hydrogel and the material surfaces. In addition, the hydrogel itself possesses soft and deformable characteristics, allowing it to conform well to substrates with different surface morphologies and form close interfacial contact, which further improves its adhesive performance. Furthermore, lap-shear measurements were performed on various substrates, including iron, copper, glass, plastic, and paper (Figure S5). The corresponding shear strengths were 30.2 ± 0.8, 30.4 ± 1.5, 49.6 ± 3.1, 25.1 ± 1.9, and 35.1 ± 3.6 kPa, respectively. These results demonstrate that CH-2 exhibits consistently strong adhesion across a wide range of substrates. Notably, benchmarking against previously reported systems reveals that CH-2 achieves superior adhesive strength (Table S3) [44,45,46,47,48,49,50,51]. These results demonstrate that the CH-2 hydrogel not only exhibits excellent mechanical properties and electrical conductivity but also possesses strong adhesion to a wide range of substrates. Such multifunctional characteristics make it highly promising for applications in wearable strain sensors, flexible electronic devices, and human motion monitoring systems.

## 4. Conclusions

In this study, a series of conductive hydrogels were successfully designed and fabricated through the copolymerization of AAM and MBAA, combined with chemically modified PVA and the conductive copolymer P(EDOT-*co*-Th). This strategy enabled the construction of composite hydrogel materials with excellent mechanical properties and electrical conductivity. The FTIR analysis confirmed the presence of characteristic absorption peaks corresponding to –OH/–NH_2_, C=O, C=C, C–S, and C–O functional groups, indicating that all components were successfully integrated into the hydrogel network structure. Mechanical property tests demonstrated that the CH-2 hydrogel exhibited the best overall performance in terms of elongation and toughness. The EIS results revealed that the hydrogels possessed stable electrical conductivity, while the LED conductivity test further confirmed that CH-2 could effectively transmit electrical current. Moreover, even after mechanical damage and subsequent reconnection, the hydrogel was still able to maintain its conductive function, demonstrating a certain degree of conductive recoverability. In terms of strain-sensing performance, the CH-2 hydrogel exhibited stable and repeatable resistance responses within a strain range of 10–200%. The sensor also maintained good signal stability under different strain rates and long-term cyclic testing conditions. In addition, the CH-2 hydrogel demonstrated excellent adhesion to various substrates, including glass, metals, rubber, and plastics. The CH-2 conductive hydrogel combines high stretchability, stable electrical conductivity, and strong adhesion properties, demonstrating significant potential for applications in wearable strain sensors and flexible electronic devices.

## Data Availability

The original contributions presented in this study are included in the article/Supplementary Material. Further inquiries can be directed to the corresponding author.

## Supplementary Materials

The following supporting information can be downloaded at: https://www.mdpi.com/article/10.3390/mi17050603/s1, Table S1. Mechanical properties of hydrogels; Table S2. Electrical properties of hydrogels; Table S3. Performance comparison of CH-2 hydrogel with reported literature; Figure S1. Tensile stress–strain curves of CH-0 (red), Control-1 (green), and Control-2 (blue) hydrogels; Figure S2. SEM images of (**a**) CH-1, (**b**) CH-2, (**c**) CH-3, and (**d**) CH-4 hydrogels; Figure S3. LED Experiment for Control-1; Figure S4. (**a**) Strain-dependent behavior of G′ (solid) and G″ (open) for CH-2. (**b**) Cyclic response of G′ (solid) and G″ (open) for CH-2 under alternating step-strain conditions; Figure S5. Shear strength of CH-2 hydrogel measured on iron (olive), copper (red), glass (orange), plastic (magenta), and paper (blue) substrates.

## Abbreviations

The following abbreviations are used in this manuscript:

AAM	Acrylamide
MBAA	*N*,*N*′-Methylenebisacrylamide
PVA	Poly(vinyl alcohol)
P(EDOT-*co*-Th)	Poly(3,4-ethylenedioxythiophene-*co*-thiophene)
FTIR	Fourier-transform infrared spectroscopy
EIS	Electrochemical impedance spectroscopy
GF	Gauge Factor
