# Supplementary material for: Optimization and Characterization of P(EDOT-co-Th)-Incorporated Poly(acrylamide)/Poly(vinyl alcohol) Conductive Hydrogels
Source: Micromachines (Basel). 2026 May 14;17(5):603. doi: 10.3390/mi17050603 (PMC13209066; doi:10.3390/mi17050603)
Supplement: Supplementary file 1 [file micromachines-17-00603-s001.zip › micromachines-4249570-supplementary.pdf]

micromachines

Supplementary Materials

Optimization and Characterization of P(EDOT-*co*-Th)-Incorporated Poly(acrylamide)/Poly(vinyl alcohol) Conductive Hydrogels

Kai-Wei Huang [1,†], Chun Hao Wang [2,†], Chien-Yin Lin [1], Rajan Deepan Chakravarthy [3], Hsin-Yu Liu [1], Yu-Hsu Chen [4], Mei-Yu Yeh [1,*] and Hsin-Chieh Lin [3,5,*]

1 Department of Chemistry, Chung Yuan Christian University, Taoyuan 320314, Taiwan
2 Neurosurgical Department, Taoyuan General Hospital, Ministry of Health and Welfare, Taoyuan 330215, Taiwan
3 Department of Materials Science and Engineering, National Yang Ming Chiao Tung University, Hsinchu 300093, Taiwan; deepannycu@gmail.com
4 Department of Orthopedic Surgery, Taoyuan General Hospital, Ministry of Health and Welfare, Taoyuan 330215, Taiwan; magister.yuhsu@gmail.com
5 Center for Intelligent Drug Systems and Smart Bio-Devices (IDS²B), National Yang Ming Chiao Tung University, Hsinchu 30068, Taiwan
* Correspondence: myyeh@cycu.edu.tw (M.-Y.Y.); hclin45@nycu.edu.tw (H.-C.L.)
† The authors contributed equally to this work.

Preparation of Control Hydrogels

Control samples were prepared to elucidate the roles of the conductive copolymer and polymer matrix components. For CH-0, acrylamide (AAm, 1.13 g) was dissolved in 2.5 mL of deionized water, followed by the addition of *N*,*N*′-methylenebisacrylamide (MBAA, 2.50 mg) and P(EDOT-*co*-Th) copolymer (5.00 mg), without the incorporation of APVA. The resulting solution was stirred at room temperature for 5 min to ensure homogeneity. Subsequently, potassium persulfate (3.75 mg) was added as the initiator, and the reaction mixture was heated to 70 °C and maintained for 2 h to obtain the CH-0 hydrogel. For comparison, Control-1 was prepared using the same composition and procedure as CH-2, except that the P(EDOT-*co*-Th) copolymer was omitted. Control-2 was also synthesized following the CH-2 formulation; however, APVA was replaced with unmodified poly(vinyl alcohol) (PVA). These control hydrogels were used to systematically evaluate the contributions of the conductive component and polymer modification to the overall performance of the hydrogels.

Rheological measurements

Rheological characterization was carried out using a TA Instruments rheometer (DHR-1, New Castle, DE, USA) with a parallel-plate geometry. Measurements were performed using a 20 mm diameter upper plate with a fixed gap of 1 mm. Dynamic oscillatory frequency sweep tests were conducted to evaluate the storage modulus (G′) and loss modulus (G″) as functions of angular frequency (ω). The frequency range spanned from 0.1 to 100 rad/s at a constant strain of 1% and a temperature of 25 °C. In addition, strain-dependent oscillatory measurements were performed by

varying the applied strain from the linear viscoelastic region (γ = 10%) to large deformation (γ = 2000%) to assess the structural stability and nonlinear response of the hydrogels.

Table S1. Mechanical properties of hydrogels.

Hydrogel	Tensile stress (kPa)	Strain (%)
CH-0	18.9 ± 0.3	146 ± 19
CH-1	34.4 ± 1.1	926 ± 35
CH-2	28.4 ± 0.4	2037 ± 61
CH-3	33.1 ± 0.6	736 ± 57
CH-4	27.1 ± 1.3	614 ± 83
Control-1	26.2 ± 0.4	799 ± 28
Control-2	29.3 ± 0.7	632 ± 52

Number of replicates= 3

Table S2. Electrical properties of hydrogels.

Hydrogel	Resistivity (Ω·m)	Conductivity (S/m)
CH-1	0.1875	5.33
CH-2	0.2250	4.44
CH-3	0.2750	3.64
CH-4	0.2625	3.81

Table S3. Performance comparison of CH-2 hydrogel with reported literature.

Hydrogel	Strain (%)	Conductivity (S/m)	Adhesive Strength (kPa)	Ref
PVA/SA/PEDOT:PSS/H_2SO_4	209	0.0487	/	S1
Laponite/PEDOT:PSS/P(OEGMA-co-DEGMA)	450	0.07	5	S2
PSS/DA/PP/HA	470	0.97	13.3	S3
CNC/PAA/Liquid Metal/Mxene/LiCl	1381	3.17	24.72	S4
PVA/TEMPO-oxi. cellulose nanofiber/EG/$ZnSO_4$	868.2	3.32	/	S5
PVA/PAA/PAAm/CNC/TA/Al^{3+}	765	2.195	16.4	S6
N-isopropyl acrylamide/Aac/Mxene/Fe^{3+}	1400	0.233	12.69	S7
Gelatin-Alginate-PNIPAM/Fe^{3+}/PEGDA/$(NH_4)_2SO_4$	250	1.78	16.61	S8
CH-2	2037	4.44	25.1~49.6	This work

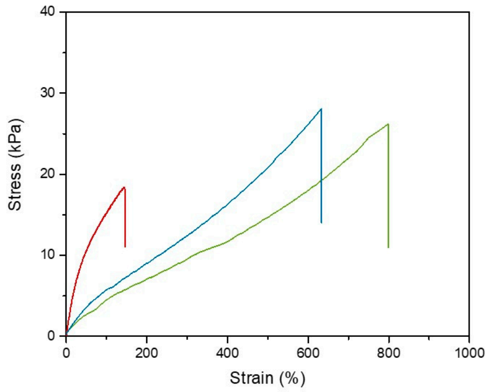

Figure S1. Tensile stress–strain curves of CH-0 (red), Control-1 (green), and Control-2 (blue) hydrogels.

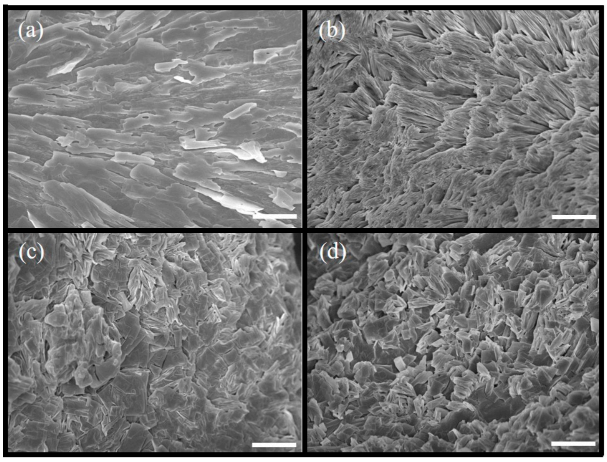

Figure S2. SEM images of (a) CH-1, (b) CH-2, (c) CH-3, and (d) CH-4 hydrogels.

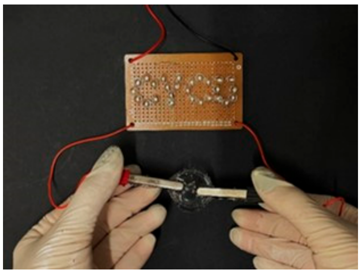

Figure S3. LED Experiment for Control-1.

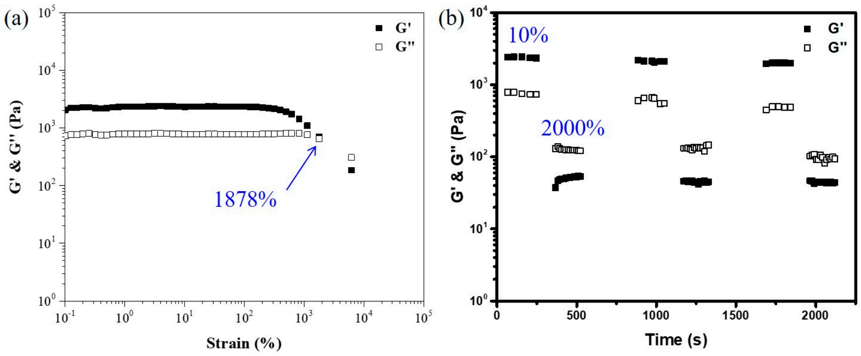

Figure S4. (a) Strain-dependent behavior of G′ (solid) and G″ (open) for CH-2. (b) Cyclic response of G′ (solid) and G″ (open) for CH-2 under alternating step-strain conditions.

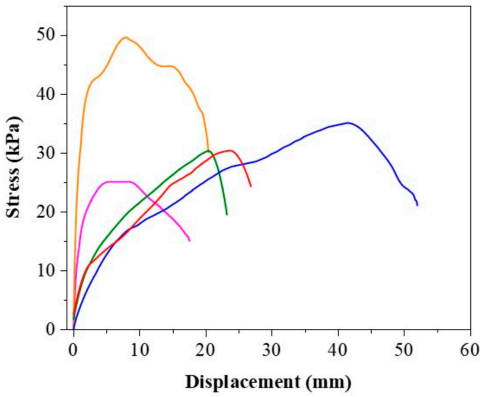

Figure S5. Shear strength of CH-2 hydrogel measured on iron (olive), copper (red), glass (orange), plastic (magenta), and paper (blue) substrates.

References

[S1] Zhou, J.; Zheng, J.; Wang, C.; Fan, M.; Wang, S.; Xiong, F.; Li, Y.; Yang, C. Fabrication of high-toughness PEDOT:PSS-based conductive hydrogel strain/temperature sensors. *RSC Adv.* **2026**, *16*, 1240-1254.

[S2] Zeng, X.; Teng, L.; Wang, X.; Lu, T.; Leng, W.; Wu, X.; Li, D.; Zhong, Y.; Sun, X.; Zhu, S.; et al. Efficient multi-physical crosslinked nanocomposite hydrogel for a conformal strain and self-powered tactile sensor. *Nano Energy* **2025**, *135*, 110669.

[S3] Zeng, M.-Z.; Wei, D.; Ding, J.; Tian, Y.; Wu, X.-Y.; Chen, Z.-H.; Wu, C.-H.; Sun, J.; Yin, H.-B.; Fan, H.-S. Dopamine induced multiple bonding in hyaluronic acid network to construct particle-free conductive hydrogel for reliable electro-biosensing. *Carbohydr. Polym.* **2023**, *302*, 120403.

[S4] Tian, Y.; Zhu, Y.; Qian, K.; Miao, M.; Ye, J.; Feng, X. Liquid metal integrated cellulose nanocrystal/polyacrylic acid dual-network hydrogel towards high-performance wearable sensing and electromagnetic interference shielding. *J. Mater. Sci. Technol.* **2026**, *251*, 124-134.

[S5] Guo, Z.; Wang, M.; Li, T.; Xiao, S.; Zhou, M.; Li, Y.; Xia, C.; Chen, C.; Fu, Q.; Wang, Q.; et al. Tough, electron-ion conductive, and anti-freezing cellulose nanofiber reinforced hydrogels inspired by honeycomb as flexible wearable sensors for intelligent human health monitoring. *Chem. Eng. J.* **2026**, *532*, 174479.

[S6] Yang, B.; Jiang, L.; Luo, S.; Yao, Y.; Cao, Y.; Li, Y. Biomimic Conductive Hydrogel Based on Polyphenol-Modified Cellulose Nanocrystals for Flexible Mechano-sensors. *ACS Appl. Mater. Interfaces* **2026**, *18*, 5835-5848.
[S7] Zhang, Y.; Pan, W.; Dong, Y.; Ding, J.; Xu, L. Stretchable, fast response and adhesive MXene-based hydrogels for wearable strain sensor. *Compos. Commun.* **2025**, *53*, 102245.
[S8] Li, S.; Wang, N.; Zhan, S.; Sheng, L.; Wang, H.; Fu, Y.; Wang, B.; Liu, C.; Yang, H. Y. Dynamic hofmeister effect-engineered thermosensitive ionic conductive hydrogel with 3D plasticity and environmental adaptability for wearable strain sensor. *J. Colloid Interface Sci.* **2026**, *711*, 140102.
